# Biofortification of wheat grain with iron and zinc: integrating novel genomic resources and knowledge from model crops

**DOI:** 10.3389/fpls.2014.00053

**Published:** 2014-02-21

**Authors:** Philippa Borrill, James M. Connorton, Janneke Balk, Anthony J. Miller, Dale Sanders, Cristobal Uauy

**Affiliations:** ^1^John Innes CentreNorwich, UK; ^2^School of Biological Sciences, University of East AngliaNorwich, UK; ^3^National Institute of Agricultural BotanyCambridge, UK

**Keywords:** nutritional enhancement, cereals, transgenic, genomics, model to crop

## Abstract

Wheat, like many other staple cereals, contains low levels of the essential micronutrients iron and zinc. Up to two billion people worldwide suffer from iron and zinc deficiencies, particularly in regions with predominantly cereal-based diets. Although wheat flour is commonly fortified during processing, an attractive and more sustainable solution is biofortification, which requires developing new varieties of wheat with inherently higher iron and zinc content in their grains. Until now most studies aimed at increasing iron and zinc content in wheat grains have focused on discovering natural variation in progenitor or related species. However, recent developments in genomics and transformation have led to a step change in targeted research on wheat at a molecular level. We discuss promising approaches to improve iron and zinc content in wheat using knowledge gained in model grasses. We explore how the latest resources developed in wheat, including sequenced genomes and mutant populations, can be exploited for biofortification. We also highlight the key research and practical challenges that remain in improving iron and zinc content in wheat.

## INTRODUCTION

All living organisms require essential mineral micronutrients to maintain metabolism and humans obtain these from their diet (Welch and Graham, 2004). However staple grains such as wheat often contain suboptimal quantities of micronutrients, especially iron (Fe) and zinc (Zn), and most of this content is removed by milling. In regions where the human diet consists mainly of cereals this leads to deficiencies in micronutrients. The World Health Organization estimates that approximately 25% of the world’s population suffers from anemia (WHO, 2008), and that Fe-deficiency anemia led to the loss of over 46,000 disability adjusted life years (DALYs) in 2010 alone (Murray and Lopez, 2013). An estimated 17.3% of people worldwide are at risk of inadequate Zn intake (Wessells and Brown, 2012) and Zn-deficiency leads to estimated annual deaths of 433,000 children under the age of five (WHO, 2009).

There are many possible strategies to improve micronutrient intake in the human diet including dietary diversification, mineral supplementation and post-harvest food fortification. However, these strategies depend on continued investment and infrastructure, and current levels of post-harvest fortification of Fe are often inadequate (White and Broadley, 2009; Gomez-Galera et al., 2010; Hurrell et al., 2010). Biofortification circumvents these problems by improving the micronutrient content of the crops themselves by increasing mineral levels and bioavailability in the edible parts. Improving crop varieties by either conventional breeding or transgenic methods has the advantage that once the initial research and development is completed, the benefits from these nutritionally-enhanced crops will be sustainable with little further investment (Gomez-Galera et al., 2010).

Many studies have shown that there is a wide variation in grain Fe and Zn concentrations in wild relatives of modern wheat and the concentrations found can significantly exceed those found in modern elite cultivars (Cakmak et al., 2000; Monasterio and Graham, 2000). This natural variation can be utilized to biofortify wheat for Fe and Zn, such as has been achieved using the transcription factor *NAM-B1 *(Uauy et al., 2006) which was originally identified for increasing protein content in wild emmer (*Triticum turgidum* ssp *dicoccoides*). In near isogenic lines the presence of *NAM-B1 *increased Fe and Zn grain concentrations by 18 and 12%, respectively, (Distelfeld et al., 2007). This gene is being widely used in breeding programes across several continents (Kumar et al., 2011; Randhawa et al., 2013; Tabbita et al., 2013).

Recent technological developments present new opportunities that can complement natural variation and genome-wide association studies, and lead to faster improvements in Fe and Zn grain content. Therefore here we focus on how the dramatic increase in wheat genomic sequence availability combined with functional genomic approaches can be used to their fullest potential to engineer new varieties of wheat with improved Fe and Zn content.

## ADVANCES IN WHEAT RESOURCES APPLICABLE TO IMPROVING FE AND ZN GRAIN CONTENT

To date, molecular breeding in bread wheat has been hindered by its large genome size (16 Gb: five times that of humans), its polyploid nature (tetraploid pasta and hexaploid bread wheat), and the high nucleotide similarity between these genomes (>95% similar in genes). However, recent advances in technology will greatly increase the rate of discovery and functional characterization of wheat genes, and provide the tools with which to deploy this knowledge into improved varieties. Some of these advances are outlined below.

### GENOME AND GENE SEQUENCE AVAILABILITY

In the last 5 years the amount of publicly available wheat genomic sequence has massively expanded. The International Wheat Genome Sequencing Consortium has coordinated the purification of individual chromosome arms using flow sorting (Safar et al., 2004) followed by shotgun sequencing and assembly into contigs of an average size of 2.5 kb. These genome-specific contigs have recently been released in *EnsemblPlants* allowing wheat researchers to separate and distinguish the homoeologous genomes for the first time. Physical maps of BAC libraries made from these purified chromosome arms are being constructed (Paux et al., 2008) to generate a high quality reference. A complementary strategy, whole genome shotgun (WGS) sequencing, has been used to generate a 5x coverage of the wheat genome, using orthologous sequences from multiple grasses to guide assembly (Brenchley et al., 2012). Draft sequences for the A and D genome progenitors, *T. urartu* (Ling et al., 2013) and *Aegilops tauschii* (Jia et al., 2013), were also created using a WGS approach followed by *de novo* assembly. Combining the WGS sequencing with the physical map strategy is leading to an unprecedented wealth of genomic information and will ultimately lead to a reliable reference sequence for polyploid wheat.

In parallel, the ability to access genic sequence through RNA-seq and exome capture (Saintenac et al., 2011; Trick et al., 2012; Winfield et al., 2012) is enabling the identification of single nucleotide polymorphisms and the development of publicly available genome-specific markers for genetic mapping in polyploid wheat (Wilkinson et al., 2012; Allen et al., 2013). Recently a comprehensive set of homoeolog-specific gene models for polyploid wheat has been published (Krasileva et al., 2013). In short, wheat researchers now have access to genome-specific contig assemblies (albeit partial and fragmented), draft reference genomes, gene models and large SNP datasets. Together, these tools should enable more precise mapping and deployment of grain Fe and Zn traits through marker assisted selection.

### NOVEL EXPERIMENTS USING SEQUENCE DATA RESOURCES

The wealth of sequence data, together with the reduced cost of sequencing, allows new ways of investigating gene function related to grain Fe and Zn. For example, RNA-seq was applied to identify differentially expressed genes in lines with reduced expression of *NAM *genes (Cantu et al., 2011). Many classes of genes including transporters, hormone regulated genes and transcription factors were identified. This study generates leads for investigations into the early stages of senescence and nutrient remobilisation that relate directly to micronutrient content in wheat grains. The differentially expressed *NAM*-regulated genes can now be further pursued through the reverse genetic resources available in wheat (see below). The refinement of methods to analyze RNA-seq data (Duan et al., 2012) together with homoeolog-specific gene models will provide increased resolution to transcriptome studies as reads can be assigned more accurately to specific genomes (Krasileva et al., 2013).

### REVERSE GENETIC MUTANT RESOURCES

The major advances and cost reduction in sequencing technology has also created the opportunity to characterize existing chemically mutagenised populations (Uauy et al., 2009; Sestili et al., 2010) for rapid discovery of mutants in specific genes. The newly developed gene models (Krasileva et al., 2013) are being combined with exome capture approaches to enrich for protein-coding genes in both tetraploid and hexaploid mutant populations. Over 3,000 individuals will be sequenced and mutations identified and organized for online access (Uauy and Dubcovsky, personal communication). Therefore in the very near future, researchers will be able to order mutants in their gene of interest through a simple *in silico* search as is standard in many model species. The availability of such resources will allow faster characterization of gene function in wheat and will provide valuable alleles for breeding.

### TRANSGENIC METHOD IMPROVEMENTS

Producing transgenic wheat has previously been a major bottleneck in investigating gene function. The efficiency of wheat transformation still lags behind the efficiency of barley transformation but it is constantly improving and a wide range of promoters is available to target transgene expression to particular tissues or developmental stages (Harwood, 2012). In addition, high-throughput *Agrobacterium*-mediated transformation of wheat is now possible through a patented technology (PureIntro; WO 95/06722) from Japan Tobacco which has been licensed to several institutions and delivers transformation efficiencies above 30%. However challenges still remain. These relate primarily to costs and the ability to produce genotype-independent transformation protocols, since most reports utilize Bobwhite or Fielder which are not suitable for commercialisation of transgenic wheat (Li et al., 2012). The ability to transform any cultivar of wheat, at a reasonable price, would allow transformation into elite lines which would speed up breeding programes and also allow research to be carried out in a more appropriate background.

## TRANSFERRING MODEL CROP KNOWLEDGE INTO WHEAT

### THE PATHWAYS FROM THE ROOTS TO THE GRAIN AND THE IMPORTANCE OF BIOAVAILABILITY

**FIGURE 1:**
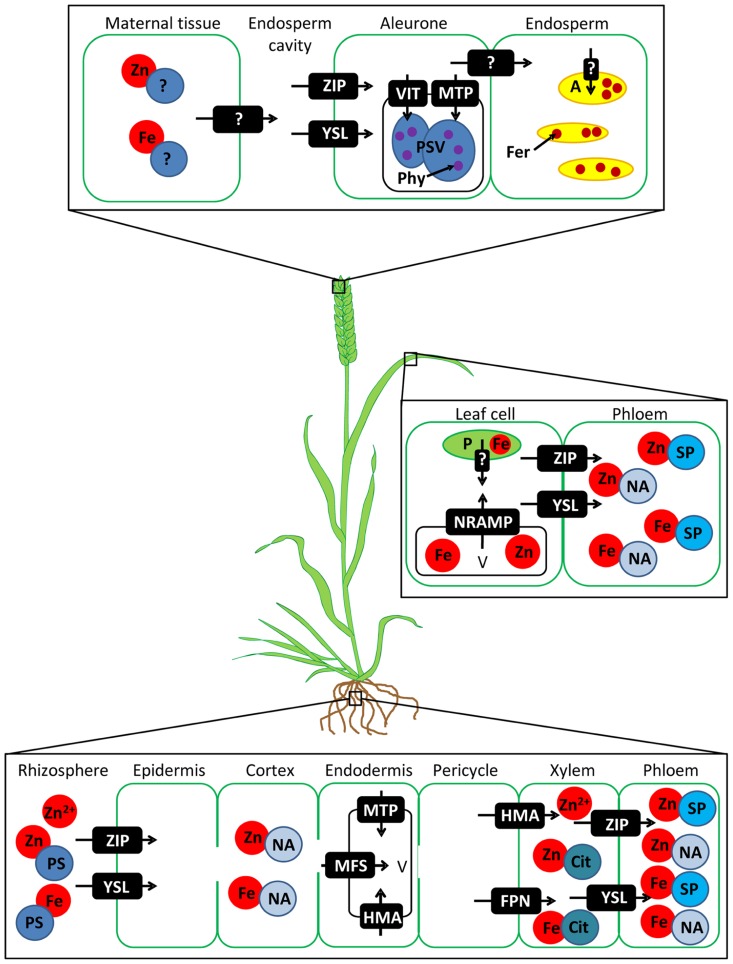
**Simplified proposed pathway for Fe and Zn uptake and translocation to the grain in wheat.** Putative classes of transport proteins are shown in white text and are based on evidence from other species. Question marks show unidentified transporters. Free Zn^2^^+^ and phytosiderophore (PS) -bound Fe and Zn are absorbed from the soil into root epidermal cells. Fe and Zn move via the apoplast and symplast to the pericycle, but may be sequestered en-route in vacuoles. Fe and Zn are loaded into the xylem and transferred into the phloem in the root, basal shoot or leaf tissues (not shown). Fe and Zn are remobilised from leaf cell plastids (P) and vacuoles (V) and loaded into the phloem for transport to the ear. Fe and Zn are exported from the maternal tissue into the endosperm cavity. After uptake into the aleurone layer most Fe and Zn are sequestered in protein storage vacuoles (PSVs) bound to phytate (Phy). A small proportion of Fe and Zn may enter the endosperm and be stored bound to ferritin (Fer) in amyloplasts (A). ZIP = ZRT-, IRT-like protein, YSL = yellow stripe like transporter, MFS = major facilitator superfamily transporter, MTP = metal tolerance protein, HMA = heavy metal ATPase, FPN = ferroportin, NRAMP = natural resistance-associated macrophage protein, VIT = vacuolar iron transporter, NA = nicotianamine, Cit = citrate, SP = small proteins.

Much work has been carried out to understand the distinct routes Fe and Zn take to reach the grain in diploid crop species such as rice, maize, and barley. Conservation of these pathways between species allows predictions about Fe and Zn transport in wheat where less is known. Recent reviews have covered the pathways in model crops extensively (Palmgren et al., 2008; Curie et al., 2009; Conte and Walker, 2011; Waters and Sankaran, 2011; White and Broadley, 2011; Borg et al., 2012; Kobayashi and Nishizawa, 2012; Lee et al., 2012; Sperotto et al., 2012; Schroeder et al., 2013) so here we will briefly outline the putative pathways in wheat and then discuss key steps to target for crop improvement (**Figure 1**).

The uptake of Fe and Zn from the soil occurs via two processes in plants: direct uptake of Fe^2^^+^ and Zn^2^^+^ by ZRT-, IRT-like proteins (ZIPs) or via secretion of phytosiderophores (PSs) which chelate Fe cations and are subsequent taken up by yellow stripe like (YSL) transporters (Sperotto et al., 2012). The chelation strategy is generally used for Fe uptake in monocots such as wheat. In many steps of Fe and Zn transport the same families of proteins are involved, however the two metals are treated separately by plants often by the involvement of different members of multi-gene families. Metal chelators such as nicotianamine (NA) are important for radial movement of Fe and Zn through the root (Rellán-Álvarez et al., 2010; Deinlein et al., 2012) and the transport of Zn into the vacuole affects overall Zn transport through the roots into the shoot (Morel et al., 2009; Haydon et al., 2012). Fe and Zn are loaded into the xylem where Zn can move as a cation or in a complex with organic acids such as citrate (Lu et al., 2013), and Fe is chelated by citrate (Rellán-Álvarez et al., 2010). Transfer from xylem to phloem can occur in the root or basal part of the shoot or during remobilisation from the leaves during grain filling and is facilitated by ZIP and YSL family proteins. In wheat all nutrients enter the grain from the phloem because the xylem is discontinuous (Zee and O’Brien, 1970). In the phloem Fe and Zn are transported as complexes with NA or small proteins. Transporters from the maternal tissue into the endosperm cavity and into the aleurone and embryo have been proposed; several are members of the ZIP, YSL, and metal tolerance protein (MTP) families (Borg et al., 2009; Tauris et al., 2009).

In wheat grain most Fe and Zn is located in the aleurone layer which is lost during milling. This problem is further compounded by the fact that Fe in these tissues is deposited mainly in protein storage vacuoles (PSVs; Regvar et al., 2011) where it is bound to phytate, which makes it poorly bioavailable to humans (Borg et al., 2009). Ferritin, which forms large Fe-rich nanoparticles, is generally regarded as a more bioavailable storage form and is present in the widely consumed endosperm amyloplasts (Balmer et al., 2006). Thus it is important to not only consider the total content of Fe and Zn in grain, but also the tissue localization and speciation (as chelates, protein particles or other), which affects their bioavailability.

Many of the steps described above have been modified by transgenic approaches in diploid crop species. We discuss below some promising studies and how this knowledge can be used to improve Fe and Zn grain content and bioavailability in wheat.

### TRANSGENIC APPROACHES IN RICE

Several studies have over-expressed genes involved in the pathway for Fe and Zn transport in rice with promising results showing increased bioavailability of Fe and no negative impact on yield. Over-expression of NA synthase (NAS) led to 2–3 fold increases in Fe and Zn content in paddy grown grain and importantly feeding this grain to anemic mice led to the recovery of normal hemoglobin and haematocrit levels within 2 weeks, whereas wild-type grain did not (Lee et al., 2009). Multiplexing genes involved at several steps has enabled even larger increases in Fe content, although bioavailability was not tested. Expressing NAS, ferritin and phytase resulted in a 6-fold increase in Fe in polished rice grains (Wirth et al., 2009). The authors suggest that the combination of these three transgenes did not significantly affect overall Fe homeostasis, shown by expression analysis of 28 endogenous rice genes in Fe-deficient and sufficient conditions (Wang et al., 2013). This suggests that a mechanism combining both increased translocation (NAS) and expanded sink strength (ferritin) could be suitable to enhance rice (and wheat) endosperm Fe content.

### APPLYING TRANSGENIC APPROACHES TO WHEAT

At present, studies in wheat are restricted to the endosperm-specific expression of wheat or soybean ferritin which led to increases in grain iron content of 1.5 to 1.9-fold and 1.1 to 1.6-fold respectively (Borg et al., 2012; Sui et al., 2012) and increasing phytase activity (Holm et al., 2002). These studies give proof of concept that grain Fe and Zn can be modified in wheat through transgenic approaches.

Using knowledge from model species, it is possible now to identify more rapidly and with higher confidence candidate genes that might play a role in Fe and Zn transport. Access to relatively complete genomic sequence for polyploid wheat will allow more comprehensive phylogenetic studies for putative wheat homologs of large gene families. For example we have used the sequences of the rice natural resistance-associated macrophage proteins (NRAMPs) to identify wheat homologs (**Table 1**). Wheat candidate genes with putative Fe and Zn transporter function inferred from phylogeny, can be taken forward and characterized in yeast mutants, with a view eventually to expressing these in transgenic wheat plants to increase vacuolar export and ultimately nutrient content in the grain.

**Table 1:** New genomic resources enable identification of NRAMP homologs in wheat.

Rice NRAMP (RAP locus ID)	Wheat homolog	Genome	Wheat sequence^a^
*OsNRAMP1* (Os07g0258400)	*TaNRAMP1*	A	7AL_4537662 (URGI)^b^
		B	7BL_6744498 (Ensembl)
		D	7DL_3317468 (Ensembl)
*OsNRAMP2* (Os03g0208500)	*TaNRAMP2*	A	4AS_5952279 (Ensembl)
		B	4BL_7000373 (Ensembl)
		D	4DL_14450878 (URGI)
*OsNRAMP3* (Os06g0676000)	*TaNRAMP3*	A	7AL_4392690 (Ensembl)
		B	7BL_6748183 (Ensembl)
		D	7DL_3360602 (Ensembl)
*OsNRAMP4* (Os02g0131800)	*TaNRAMP4*	A	6AS_4346871 (Ensembl)
		B	6BS_2318478 (Ensembl)^c^
		D	Absent
*OsNRAMP5* (Os07g0257200)	*TaNRAMP5*	A	4AS_5926812 (Ensembl)
		B	Across multiple contigs
		D	4DL_14404139 (URGI)
*OsNRAMP6* (Os01g0503400)	*TaNRAMP6*	A	Across multiple contigs
		B	Across multiple contigs
		D	Across multiple contigs
*OsNRAMP7* (Os12g0581600)	*TaNRAMP7*	A	5AS_1501999 (Ensembl)^b^
		B	5BS_2288821 (Ensembl)
		D	5DS_2767814 (Ensembl)
–-	*TaNRAMP8*	A	4AL_7173573 (URGI)
		B	4BS_3944622 (Ensembl)
		D	4DS_2292562 (Ensembl)

a International wheat genome sequencing consortium chromosome-arm survey sequences are available at *EnsemblPlants* (Ensembl; ) and at Unité de Recherche Génomique Info (URGI; )

b partial sequence

c premature termination codon

Work in rice has shown that multiplexing genes can further increase gains and this strategy could also be suitable for wheat. An analogous approach which would act to regulate several steps of the Fe and Zn uptake pathways would be to engineer transcriptional regulators to enhance movement and uptake into grains. For example the *NAM-B1* transcription factor provides an entry point to increase Fe, Zn, and protein content: with greater understanding of its targets, and which transport steps are key control points, we could engineer expression patterns, downstream targets, or binding specificities to improve nutrient content in the grain. The lack of genomic resources in wheat prompted initial studies on *NAM-B1* to focus on rice, but these attempts failed since the orthologous rice *NAM* gene affects anther dehiscence rather than mineral remobilisation (Distelfeld et al., 2012). The advent of new technologies and genomic resources now allow these questions to be addressed directly in wheat. In addition to RNA-seq (Cantu et al., 2011), we have developed transgenic lines with epitope-tagged NAM proteins to perform chromatin immuno-precipitation followed by sequencing (ChIP-seq) to identify direct targets of NAM (Borrill and Uauy, unpublished data). The availability of gene models and genomic sequence (including promoter regions) now makes this a feasible undertaking in wheat.

### CHALLENGES IN TRANSFERRING KNOWLEDGE TO WHEAT

As illustrated by *NAM* in rice and wheat it is important to note that the precise mechanisms of transport and its regulation differ between species even within monocots, so not all knowledge can be directly translated. It has been proposed that Zn transport to the grain in wheat is constrained by two major bottlenecks; the root–shoot barrier and grain filling (Palmgren et al., 2008). However in rice these bottlenecks are reduced (Stomph et al., 2009) as shown by the storage of excess Zn in shoots as well as roots (Jiang et al., 2008), and the ability of rice to load Zn from the xylem into the grain without transfer to the phloem (Zee, 1971), which constitutes a limiting step in wheat. Fe transport in rice and other monocots also differs, for example rice uses both secretion of PSs and direct uptake of Fe from the soil (Bughio et al., 2002; Inoue et al., 2009; Nozoye et al., 2011), whereas barley and maize only absorb Fe via PSs (Römheld and Marschner, 1986; Zaharieva and Römheld, 2000; Murata et al., 2006; Walker and Connolly, 2008). These challenges are compounded by the fact that several fundamental questions remain unanswered even in model species. The specificity of many transporters is not fully characterized and the control of flux through pathways requires further investigation. Additionally, the relative contribution of sink/source strength and the possible effects of manipulating individual metals on total grain metal composition are not well understood.

## CONCLUSIONS AND FUTURE DIRECTIONS

Our ability to carry out basic research in wheat will be extremely important to build upon and move beyond research in model species. This will be greatly advanced by recent developments in genomic resources, mutant catalogs and transgenic methods. Once genes to improve nutrient content have been identified, they will need to be transferred into agriculturally relevant wheat varieties and assessed for agronomical traits such as yield and disease resistance which are the main drivers for adoption of novel varieties by farmers.

Changes to agricultural conditions in the future will also impact upon the deployment of biofortified wheat varieties. Rising atmospheric carbon dioxide (CO_2_) concentrations may lead to reduced grain micronutrient content, especially Fe, as has been shown by free air CO_2_ enrichment studies (Hogy et al., 2009; Fernando et al., 2012). Additionally lower levels of fertilizer may be used in the future due to budgetary and legal constraints. This may present a challenge to improve Fe and Zn grain concentrations because lower application of nitrogen fertilizer correlates to lower Fe and Zn grain concentrations (Cakmak et al., 2010). In addition, the drive for higher yields is usually accompanied by a dilution effect of minerals due to the additional grain starch accumulation. This scenario suggests that scientists and breeders will need to work ever more closely to achieve not just maintenance, but the required increased grain Fe and Zn contents.

Despite these challenges we believe that wheat researchers now have access to the tools and resources required to make significant improvements to Fe and Zn content in wheat grain and to bring these improved varieties to the field. These new varieties could make an important contribution to improving the health of millions of people worldwide to avoid Fe and Zn malnutrition which still affects over 25% of the global population.

## AUTHOR CONTRIBUTIONS

Philippa Borrill and Cristobal Uauy wrote the manuscript; James M. Connorton, Janneke Balk, Anthony J. Miller, Dale Sanders contributed corrections and suggestions; Philippa Borrill, James M. Connorton, Cristobal Uauy analyzed the wheat genome sequence data for **Table 1**. Philippa Borrill, Janneke Balk, Anthony J. Miller, Dale Sanders, Cristobal Uauy conceived the perspective. All authors read and approved the final manuscript.

## Conflict of Interest Statement

The authors declare that the research was conducted in the absence of any commercial or financial relationships that could be construed as a potential conflict of interest.
